# An Integrated IoT Architecture for Smart Metering Using Next Generation Sensor for Water Management Based on LoRaWAN Technology: A Pilot Study

**DOI:** 10.3390/s20174712

**Published:** 2020-08-20

**Authors:** Vlastimil Slaný, Adam Lučanský, Petr Koudelka, Jan Mareček, Eva Krčálová, Radek Martínek

**Affiliations:** 1 Department of Agricultural, Food and Environmental Engineering, Mendel Univerzity in Brno, 61300 Brno, Czech Republic; xlucans1@mendelu.cz (A.L.); petr.koudelka@mendelu.cz (P.K.); jan.marecek@mendelu.cz (J.M.); eva.krcalova@mendelu.cz (E.K.); 2 Department of Cybernetics and Biomedical Engineering, VSB—Technical University of Ostrava, 70800 Ostrava-Poruba, Czech Republic; radek.martinek@vsb.cz

**Keywords:** smart metering, LoRaWAN, smart water, modularity, water management

## Abstract

This pilot study focuses on the design, implementation, optimization and verification of a novel solution of smart measuring of water consumption and crisis detection leading to a smart water management platform. The system implemented consists of a modular IoT platform based on a PCB (Printed Circuit Board) design using the M2.COM standard, a LoraWAN modem and a LoraWAN gateway based on the Raspberry Pi platform. The prototype is modular, low-cost, low-power, low-complex and it fully reflects the requirements of strategic technological concepts of Smart City and Industry 4.0, i.e., data integration, interoperability, (I)IoT, etc. The study was produced in cooperation with M.I.S Protivanov and VODARENSKA AKCIOVA SPOLECNOST, a.s. (industry partners distributing drinking water in the Olomouc and South-Moravian regions) to depict the current situation in the Czech Republic, characterized by extreme weather fluctuations and increasingly frequent periods of drought. These drinking water distributors are also constantly placing new demands on these smart solutions. These requirements include, above all, reliability of data transmission, modularity and, last but not least, low cost. However, smart water management (water consumption, distribution, system identification, equipment maintenance, etc.) is becoming an important topic worldwide. The functionality of the system was first verified in laboratory conditions and, then, in real operation. The study also includes checking signal propagation in the municipal area of the village of Zdarna, where the radius of the proposed measuring system was tested. A laboratory test with simulation of water leakage is also part of this work. Subsequently, the system was tested in a residential unit by means of water leakage detection using the MNF method (minimum night flow); the detection success rate was 95%.

## 1. Introduction

**Figure 1:**
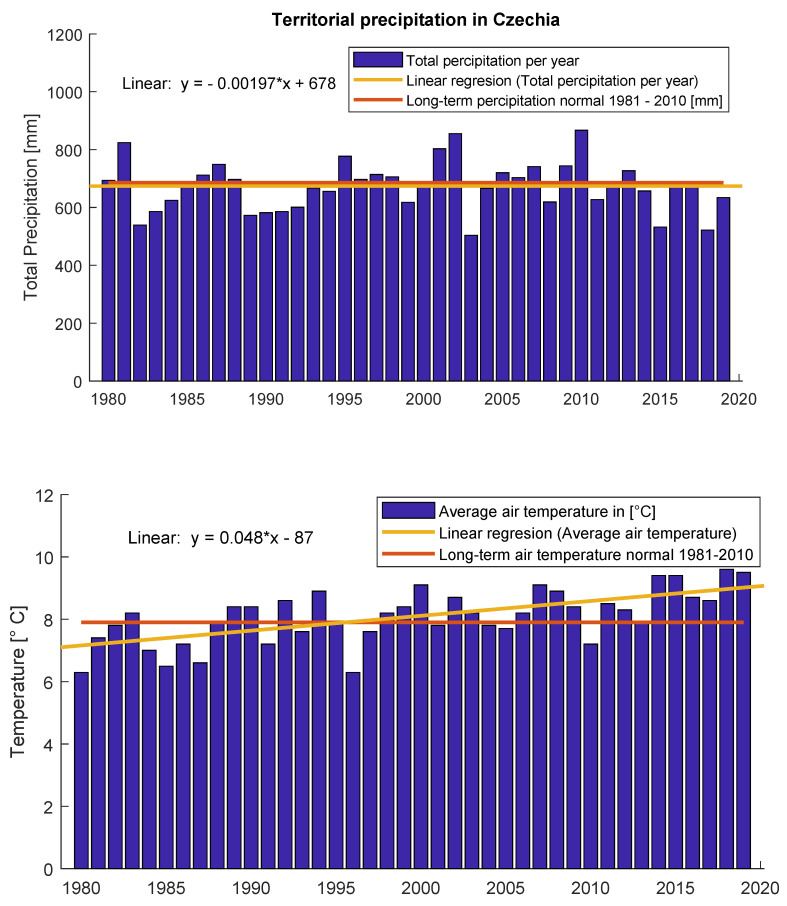
Territorial precipitation and territorial temperature in Czechia [4]

The topic of water management has gained prominence recently because of extreme weather in the Czech Republic [1], which is currently mapped by the INTERSUCHO project [2]. The Aqueduct prediction model [3] expects two or three times higher water stress until 2030 in almost half of the territory of the Czech Republic. Figure 1 shows the progression of annual precipitation and the average temperature in the Czech Republic.

However, Ref. [5,6,7] proved that extreme weather fluctuations and drought are not problems of only the Czech Republic. They are becoming a global issue with no promise of improvement in the future [8] along with the associated intelligent water management (water consumption, distribution, water quality monitoring, system identification, equipment maintenance, etc.) [9,10,11]. The analysis conducted in the last few years proved that these intelligent measuring systems are highly beneficial and effective, not only in the case of involving citizens in reducing energy consumption, but also when identifying possible waste of resources [12,13]. For example, the average distribution loss in drinking water in the EU countries is 23% [14].

In the Czech Republic, a volumetric flow meter is currently most often used to measure the consumption, mainly due to its low price and good reliability. Its function is based on measuring the speed of water flow through a pipe when it is rotated by a piston or turbine. The volumetric water flow is directly proportional to the speed of rotation of the blades. Mechanical water flow meters may become clogged if the water is dirty or contains larger particles (leading to increased maintenance costs), which is their disadvantage. At low flow rates, the error rate of these water meters also increases. Recently, these mechanical flow meters have been equipped with additional electronics allowing them to read remotely. These water meters are indicated as AMR (Automatic meter reading) [15]. The number of these water meters in the Czech Republic is still low, mainly due to the purchase price paid by the end customer. Another option may be fully electronic water meters employing new measuring methods, such as electromagnetic [12], fluidic and ultrasonic meters [15]. According to Act 274/2001 Coll. each building that wants to be connected to the water supply network must be equipped with one of the aforementioned types of water meter (the water meter must be certified for the required use) [16]. Table 1 shows an overview of households connected in the Czech Republic by region. The total number of buildings connected is 94.7% with a total consumption of 609.7 million m^3^ of drinking water, when the losses reached 95 million m^3^, which is 15.8% of the total consumption (the losses are broken down by individual entities connected to the distribution network) [17]. The system monitoring consumption of drinking water has not yet been used on a larger scale in the Czech Republic. Mostly, they are just big cities where pilot projects are taking place. For example, in Prague, remote readings of water meters have been being implemented since 2013. In 2019, the number of water meters installed reached 8244, which is approximately 7.2% of the total number of 113,848 water meters (operated by Pražské vodovody a kanalizace, a.s) [18]. It is also important to state that sewage (waste water) pricing in the Czech Republic is broken down by individual entities on the basis of drinking water consumption. In the event of an accident, the entities are, therefore, charged not only for the drinking water consumed, but also for the waste water.

As for foreign companies, we can mention, for example, Thames Water supplying drinking water to the Thames Valley (in the south-east of England) and most of London. In 2013, this company came up with a program to install smart water meters. The goal of this program is to provide each of the 9 million customers with their own water meter by the end of 2030, thus allowing remote readings (except for problematic locations). This is the first smart metering project in the water industry in the United Kingdom. In 2019/20, 449,000 water meters, installed primarily in the area with the highest water leakage, were managed by this company. Thanks to this step, the year-on-year reduction in leakage is 13.8%. [19] When reducing water consumption and using this technology, we will also experience the following benefits:

- The load on the pumps will be reduced, which will be reflected primarily in longer service life and lower maintenance costs
- Better sustainability and management of current water resources (in certain parts of the year, for example, restrictions on drinking water consumption are announced when, for example, it is forbidden to water gardens, etc., with tap water)
- Early detection of unusual conditions—such as unauthorized entry into the shaft, drop in ambient temperature below the allowable value or detection of zero water consumption at a certain time (used, for example, to check senior citizens, wherein zero water consumption may mean a health issue of this person)
- Fault detection—for example, water meter failure, backflow or attempted unauthorized manipulation of the water meter.

The development of IoT (Internet of Things) technologies enables us to use smart technologies in areas yet unimaginable. Their continuous integration into daily life is associated with the broad support of Smart City projects, which use the data collected (consumption of water, gas, electricity, or street lighting) for improving the quality of life in cities [20,21,22,23]. The Smart City concept envisages the deployment of sensors at important nodes in the distribution network, which will allow us to detect the large leaks quickly [24,25].

The basic methods of water leakage detection include the AMR method (which is also included in our solution). Leakage detection is evaluated on the basis of the data read, which is then processed and evaluated [26]. Hamilton and Charalambous [27] list several other basic methods for detecting water leaks:

- Gas injection method: Is very effective in cases where acoustic methods cannot be used due to the absence of sound signals. Tracer gas, which spreads well through the pipeline and rises to the surface at the point of the defect, where it can be located with a suitable measuring device with sensitivity to this gas, is fed into the circulation system.
- Manual listening stick: Is used to listen to the leaks on fittings and to pinpoint the location of the leak. The listening stick may be made of metal, wood, or plastic.
- Hydrophones: This is a sensitive microphone that is in direct contact with water and can, therefore, capture sounds transmitted by water and not just by the pipes. Based on the analysis of the noise, a possible water leakage can be detected [28,29].
- Noise logger corelation: These devices are placed in accessible places on the pipeline, where they try to detect and evaluate the noise. The leakage is always located between two noise loggers, where the position of the fault can be calculated [30].
- Thermal imaging: They can detect temperature differences that reveal the presence of water. They are mainly used to detect cracked pipes in buildings [31,32].

However, the aforementioned methods are relatively expensive and technically demanding. In addition, they are mainly used to detect water leakage on the supplier’s side. In contrast, our solution focuses on end users.

Many types of water meters without the necessary support for remote reading are available on the market. They are designed primarily for the visual representation of water consumption when a human operator’s presence is still required. However, the Directive of the European Parliament and the Council (EU) 2018/2002 [33] sets out a new rule of obligation to install only devices that allow remote reading from 25 October 2020 [33] in order to increase energy efficiency throughout the energy chain and to provide consumers with accurate, truthful, and reliable information of their energy consumption (especially concerning the billing of heat and hot water). Devices installed before this date must be replaced no later than 1 January 2027 [33]. Nevertheless, the market does not offer an open and widely reputable communication standard for data transmission. Thus, most producers develop their own solutions with the implementation of novel communication protocols, such as LoraWAN, Sigfox, or NarrowBand IoT [13].

The study focuses on the implementation of IoT remote system for monitoring water consumption (including possible leaks) using the LoraWAN technology. The current development in the IoT area allows us to meet the ever-increasing demands on the economical use of water resources, caused by the current climate situation in the Czech republic. Since most producers must rely on signal coverage by the local providers, who charge a monthly fee for each device connected to their network, we also proposed a new low-cost modular LoraWAN gateway. The modularity, where other peripherals can be connected to our device via the integrated MikroBUS bus, whether it is another water meter or a flood sensor, is also another advantage of our system. A temperature sensor, which can be used to control the temperature in water supply shafts, is also directly integrated into the device. The system is also ready for possible replacement of the communication module) with another type (e.g., SigFox). However, this version has not been tested yet.

### Related Works and Solutions

The use of smart technologies in measuring systems is a frequent topic in the research, and many various solutions in the area of energy reading (water, gas, or electricity) are described. Jin, G. et al. [34] and Li et al. [35] proposed a system for measuring water consumption based on image recognition technology, using MCU (Microcontroller unit) STM32F103ZET6 and camera module OV7725. The original image of the water meter counter is preprocessed using greying, edge extraction, Otsu’s binarization, and tilt correction. The digits extracted in this way are then recognized by CNN (Convolutional Neural Network) using the structure of a classical two-stage target detection network [36]. The data is then transmitted to the server using the NB-IoT (NarrowBand-Internet of Things) technology. Kelnar, M. et al. [37] described the implementation of a low-cost, two-way, and fibreless optics communication system for water meter reading, which is implemented again using image recognition. Al-Ali et al. [38] proposed an IoT device determined to measure the consumption of electricity, water, and gas, providing remote access, and making the desired configuration. The data is read by the pulse output and displayed to the user with the goal to increase the outline of the consumption. The sensors are connected to the central control unit (Raspberry Pi), collecting the data required and, then, sending it to the server. Similar issues are dealt with in [13,39,40].

Besides neuron networks, ultrasound sensors provide more precise reading, especially in a low flow rate zone [41]. In [42], the measuring system based on a control unit (a centralizer), which can communicate with ultrasound sensors via Mbus or wirelessly using the LoRaWAN technology, is described. The data is available for the users in cloud storage, where it is derived from the control unit by means of a mobile network. The shortcomings of this method are discussed in [43]. Short sound path, weak echo signal, and different temperature gradient result in the decline of accuracy and stability.

Another reading method uses the pulse output. The pulse is usually a mechanical contact switching with each countdown wheel turning in the water meter. Other water meters are equipped with a magnetic needle [44], and the sensor is placed on the water meter glass. The sensor detects the needle’s magnetic field and indicates the result as the number of its rotation cycles. The pulse signal obtained can be transferred to the PLC (Programmable logic controller) and then processed.

There are several different solutions to data transmission. The most common technologies in the area of smart measuring systems and long-distance data transmission include the LoraWAN [45,46,47], Sigfox [48,49] or NB-IoT technologies [34,50]. These technologies are compared in [51], where their benefits and shortcomings are discussed. In particular, the safety of IoT networks is an important and frequently discussed issue. For example, Lin et al. [52] described the architecture, the useful technologies, and the IoT security, discussed the potential safety issues and privacy protection, and proposed possible solutions. Navarro-Ortiz et al. [53] focuses on the safety of the LoRaWAN technology and describes the possibility of its improvement on the HW level. The analysis of LoraWAN, Sigfox, and NB-IoT vulnerability is conducted in [54]. This work [55] describes the concept of Small Cell Cloud (SCC) composed of multiple Cloud-enabled Small Cells (CeSCs), which provide radio connection for mobile User Equipment (UE), such as smart-phones or wearables.

First, analysis of the available solutions of smart water management and comparison of the technologies used for remote data transmission were conducted in Section 2. Based on this review and the requirements on the system, we selected the LoraWAN technology. Section 3 describes the development of the motherboard with the M.2 module. Finally, the results of system testing under laboratory conditions with the controlled simulation of water leakage and in an apartment building, and gateway testing in real operation are shown in Section 4.

## 2. Matherial and Methods

**Figure 2:**
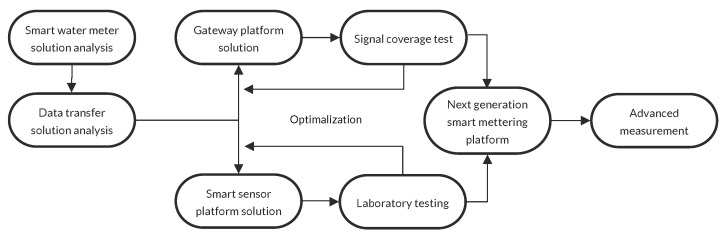
Smart water meter concept.

The entire process of the prototype design is described in Figure 2.

**Figure 3:**
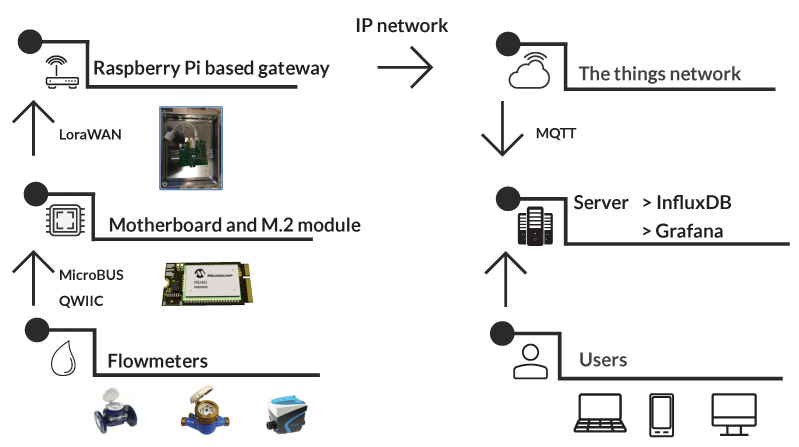
Concept of the proposed smart metering system.

The proposed platform is modular, as shown in Figure 3, and consists of two modules: (1) Carrier board and (2) M2.COM processor module with the necessary communication protocol. Both modules are described below. Due to the use of the device in water supply shafts, it is necessary to protect the device especially from high humidity. For this purpose, standardized junction boxes with IP protection index 67 are used.

When proposing the solution, the following requirements were set on the basis of cooperation with the companies MIS s.r.o. and VAS a.s.:

- Modularity between the modules connected for reading the data from the sensors and the rest of the system
- Use of exclusively standardized interfaces
- Possibility to connect other external sensors (e.g., a flood sensor) via these standardized interfaces
- DC-DC converter to the battery power supply
- Minimum battery life of two years
- Integration of the LoraWAN gateway into the data transmission system
- Data transmission interval from the device to the gateway, at least once an hour.
- Low production costs for surface deployment

Another part of the measuring chain comprises a gateway that collects the data from the individual sensors. The gateway used is based on the Raspberry Pi platform using an iC880A-LoRaWAN® Concentrator 868 MHz board by IMST (Germany).

The modular system of the proposed device comprises a front-end module with two open-collector inputs, so a magnetic reed switch and any commercially available readout attachment for flow meters can be used. The over-voltage protection and the RC filter of the inputs are set to filter pulses less than 20 ms and noise.

**Figure 4:**
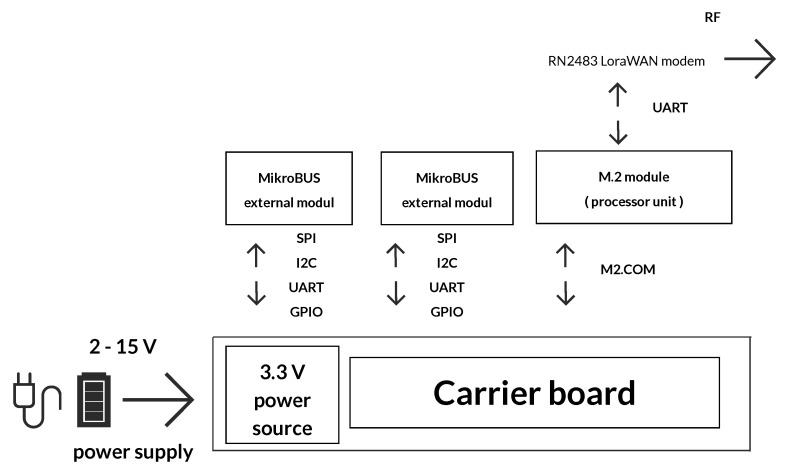
Diagram of the proposed measuring sensor.

The general diagram of the proposed measuring system is described in Figure 4.

### 2.1. Carrier Board

**Figure 5:**
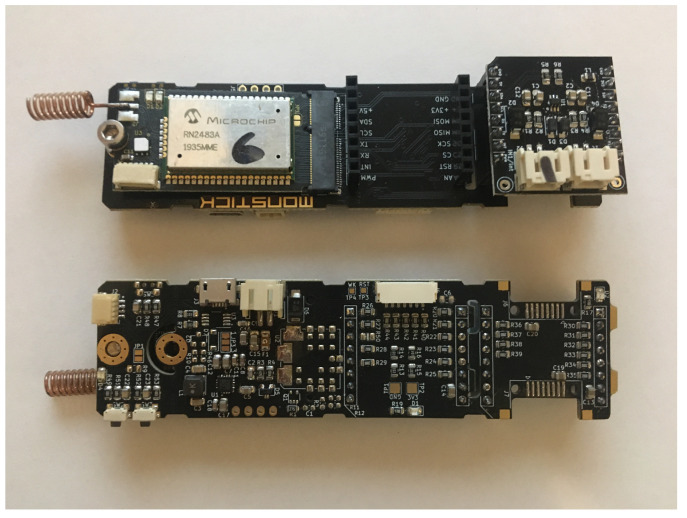
Printed Circuit Board (PCB) with M2.COM module and microBUS module for reading from pulse water meter.

As shown in Figure 4, the carrier board consists of a power connector (JST-PH2) with a power range from 2 to 15 V, an M2.COM connector, a DC-DC converter, and connectors selected depending on the interfaces. The complete design is presented in Figure 5. The power supply of the entire device is designed with regard to the secured functionality, both in a low voltage range of the LiFePO${}_{4}$ battery (2.6 V) and in a high range of 12 V of the accumulator (maximum 13 V). We assume that the maximum current on the 3.3 V branch does not exceed 200 mA. A buck-boost LTC3129 chip was used as integrated switch-mode power supply [56]. The proposed power supply circuit enables the processor module to shut down, if it is supplied externally, to prevent overcharging between these sources. This is a preparation for the M2.COM module with an external connector (USB), which will be used to power the entire device during programming.

### 2.2. M2.COM Processor Unit

**Figure 6:**
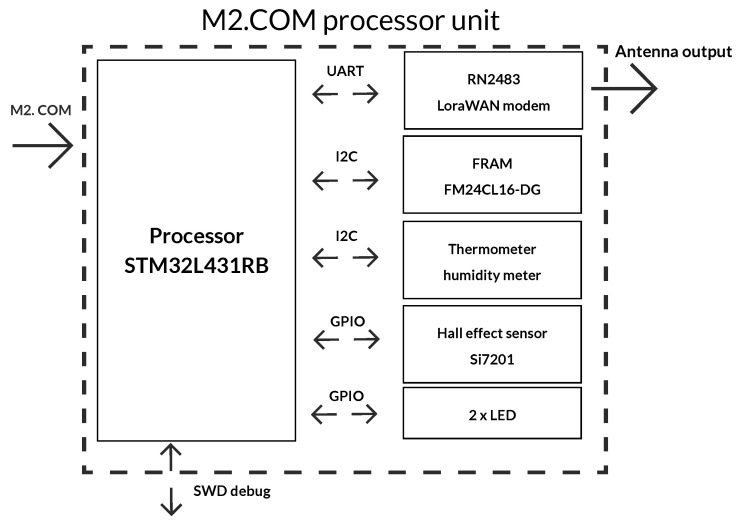
Block diagram of the processor module.

The main part of the processor unit comprises a module that includes a processor, a modem, and selected peripheries (F-RAM memory, Hall probe, and two LEDs). A block diagram of this module is shown in Figure 6. The peripheries of the microprocessor are connected to the side of the M2.COM connector, which is also used as an input for the power supply and other signals (resetting or switching off external buses). The antenna can be connected through the SMA connector with 50 Ω conduction to the LoRaWAN module. The basic properties of the STM32L431RB processor are summarized in Table 1 [57].

The I2C (Inter-Integrated Circuit) temperature and humidity sensor (Si7006-A20-IM) is placed on the top of the board. It serves for testing the communication and functionality after the assembly (the data successfully sent indicates a functional critical path). Its accuracy is determined by the producer $\pm 1{\;}^{\circ }C$ ($-10{\;}^{\circ }C$ to $85{\;}^{\circ }C$) a ±5%RH (0–90% RH) [58].

State and configuration preservation is secured by F-RAM memory, which is characterized by practically an infinite number of transcripts ($10^{14}$), thus enabling saving the current state of the water meter counter without destroying the memory cells. We choose the FM24CL16B−DG chip with the I2C interface and a capacity of 16 kbit [59]. Wireless communication and data transmission are performed via an RN2483 modem made by Microchip. This module provides the LoRaWAN protocol using a simple UART (Universal asynchronous receiver-transmitter) interface (communication with module passes on the serial port using the ASCII protocol) and enables us to use the LoRaWAN CLASS A protocol, designed for low-energy equipment [60]. The connector, 6-wire JST-PH (1 mm spacing) in the SMD (Surface Mount Device) case, was used for program tuning and recording purposes according to the DroneCode standard. Besides the power supply wire and the negative pole, there are also two SWD wires (Serial Wire Debug) and the UART.

### 2.3. Reading Module

**Figure 7:**
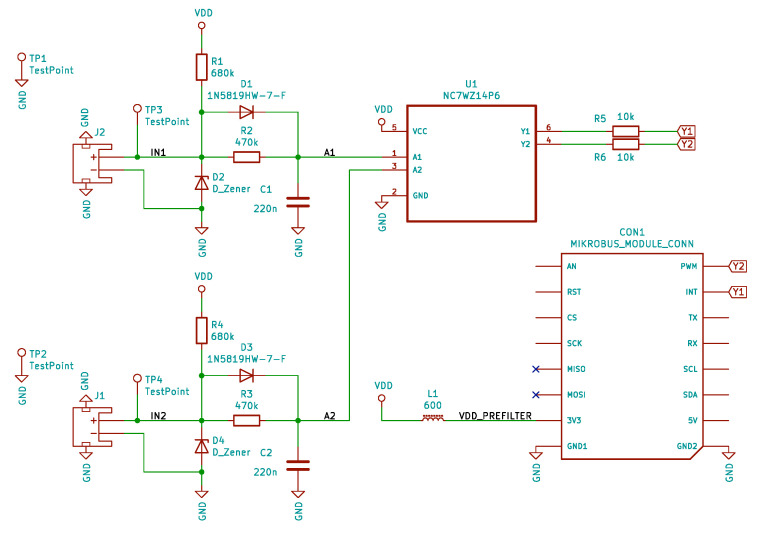
Scheme of data reading module from the selected water meters.

The proposed module shown in Figure 7 can be used to sense any switching mechanism, optionally with a phototransistor. The module consists of an RC filter connected with a diode providing time settings to stabilize output oscillation during the switching on and off process. Moreover, overvoltage protection and dual Schmitt trigger NC7WZ14P6 [61] are used. The proposed module is actually shown in Figure 5, top view, right side.

### 2.4. Firmware

The firmware for the selected STM32 microcontroller on the proposed IoT platform uses the ecosystem of Ivory and Ivory-Tower made by Galois, which is written in the Haskell functional programming language [62,63]. The advantage of this solution is the ability to quickly change the functionality based on the current requirements. The Tower framework’s main concept is a description of behaviour by Hoare monitors with synchronous typed channels that resemble a Petri net. Such a description of behaviour is then transformed into the C language along with the auxiliary code (FreeRTOS).

### 2.5. Transmission Frequency

The device contains internal F-RAM memory, where the current state of the pulse counter is stored at regular intervals to maintain its state after a power outage. This data is subsequently sent in a predefined time interval using the LoraWAN module. In our case, the time interval for sending the data is set to 1 h. The minimum interval for sending the data is 3 min.

### 2.6. Energy Consumption

For the validation and testing purpose, the device was powered from a wall outlet adapter, but the theoretical lifespan for operation using a battery (3xAA LR6 battery) is a minimum of two years with a 1 h interval of sending the data obtained (pulse counter, humidity and temperature) (calculation based on STM32CubeMX) [64]. The general consumption at 132 μA is 3.0 V.

### 2.7. LoraWAN Gateway

**Figure 8:**
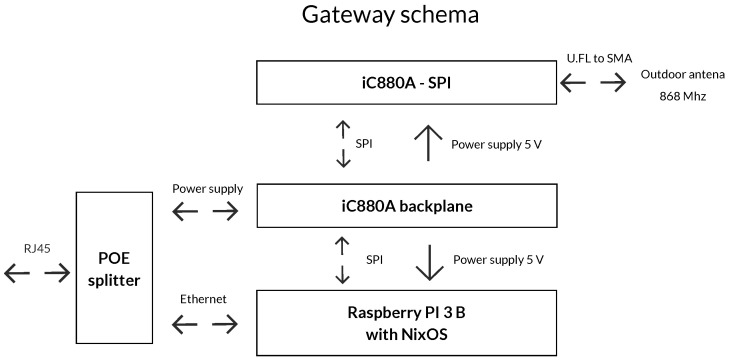
Gateway schema based on Raspberry PI and ICC880A.

The gateway is based on Raspberry PI 3 B + a platform (see Figure 8), to which an iC880A backplane is connected through an SPI (Serial Peripheral Interface) bus. The backplane is a board for connecting the iC880A-SPI to the Raspberry Pi and serves for supplying the Raspberry PI and the iC880A-SPI board itself (see Figure 6) [65,66].

The iC880A module can be used for many applications, such as Smart Metering, IoT, or M2M network building. It can receive packets from several different endpoints simultaneously, when they are sent with different SF settings, through up to 8 channels concurrently. Hence, it provides the possibility of robust communication between the gateway and a large number of endpoints at different distances. The proper function of the iC880A needs a host system (in our case Raspberry Pi 3 B combined with NixOS). This module is also connected with an external antenna using a U.FL−N converter. The technical parameters of the antenna are summarized in Table 2.

As soon as the data is received by the gateway, it is immediately sent, via the IP Network, to the server, where the data is further processed and evaluated.

#### LoraWAN Transmission Technology

The gateway, based on the LoraWAN technology, is a low-power consuming wireless network protocol proposed for low-cost and safety communication in the IoT area. LoRa is a physical layer used to establish a remote communication link, based on Chirp-Spread-Spectrum modulation, which preserves the same properties of low-power consumption as FSK (Frequency-Shift Keying) modulation, but significantly increases the possible communication range. Chirp-Spread-Spectrum is used mainly in military and space communications, due to the possible long communication distances, and is resistant to interference. LoRa is the first low-cost implementation of this technology for the commercial use [67].

Another advantage of LoRa is the possibility of flexible configuration, which enables changing the signal range by adjusting the bandwidth (BW), the spreading factor (SF), and the coding rate (CR). According to EU863−870 Industrial, Scientific, and Medical (ISM) specifications, the BW can reach values of 125 kHz, 250 kHz, and 500 kHz. The SF can be set in the range from 7 to 12, when it reaches the value of 7 for short-distance communication (up to 4 km depending on the environment) and the value of 12 for long-distance communication. The CR improves the robustness of code receiving of 4-bit data with redundancies for error correction in variants 4/5, 4/6, 4/7, or 4/8. The bit rate of LoRa is calculated according to the following equation [68,69]:

(1) $$R_{b}=SF\;\cdot \;\frac{BW}{2^{S}{}^{F}}\;\cdot \;\frac{4}{4+CR}\;[\mathrm{bit}\;s^{-1}]$$

where SF is the spreading faktor; BW is the band width; CR is the code rate.

Equation (1) shows that the *SF* is one of the dominant factors for the computation of the bit rate, which defines the physical speed of data transmission (data rate (DR), DT), as shown in Table 3. The DR in the range from 6 to 14 is not included because it is not implemented in ISM EU863−870 [69].

The nodes in the LoraWAN network are asynchronous, and the communication passes off after the data is ready to be sent, no matter whether this condition is event-driven or scheduled. The nodes often have to “wake up” in a mesh or synchronous network, such as a cellular one, synchronize with the network and check the message. This synchronization consumes a considerable amount of energy and is the main reason for shortening the battery life.

Terminal equipment is also used for different applications and has completely different requirements. Terminal equipment of class A provides two−way communication when each uplink transmission of the terminal equipment is followed by two short receiving windows for the downlink. The emitting slot of the terminal equipment is based on its own communication needs (ALOHA protocol). Hence, the terminal equipment requires downlink communication from the server shortly after it sent the uplink to the server. Any other downlink communication has to wait before the next sensor starts emitting. Terminal equipment of class B opens other receiving windows, such as the reaction on a trigger from the gateway indicating the “listening” of the terminal equipment. Terminal equipment of class C has almost continuously open receiving windows, closing only during the data transmission to the server [67,70].

The concept of the device meets the requirements for classification in Class A, especially because of the intended use in water supply shafts, where the battery is the only source of electric energy.

## 3. Results

The experimental workplace was built for primary testing, as shown in Figure 7. The closed testing circuit consisted of a water tank, a pump, and one Meinecke WS-50 water meter with an output for reed contact (when K = 1 and K = 10) and a MEDER KSK1A66-1020 reed switch. This reed contact switches when a magnetic field is present. The reed switch was soldered to the end of the two-core cable (originally one-channel video cable with a CINCH connector), and the JST-PH2 connector was added to its other side. The switch could be connected directly to the microprocessor output, but the accurate and reliable measurement requires “front—end” (see Section 2.3), which avoids interference and oscillations, and creates overvoltage protection.

**Figure 9:**
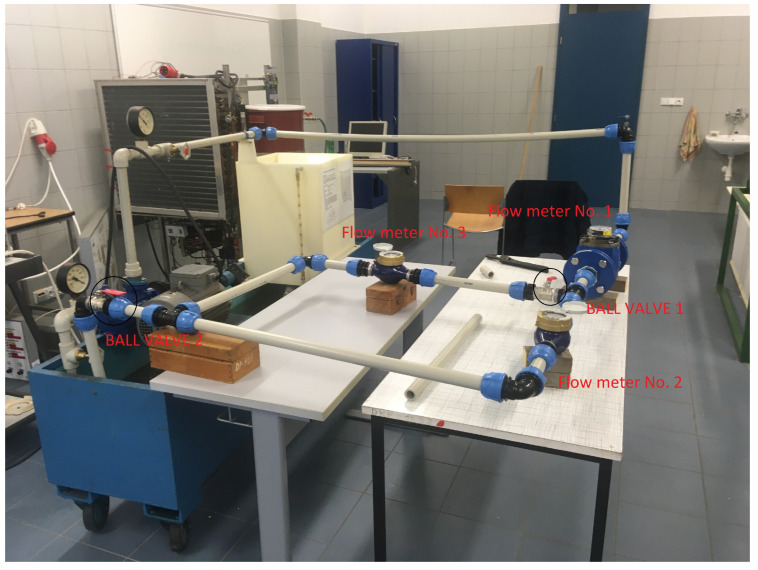
Laboratory testing circuit.

Then, two water meters, SENSUS 420 [71], equipped with pulse module output, are connected in parallel. An HRI-A (High-Resolution Interface) pulse module by SENSUS is connected to the interface described above (see Section 2.3). Moreover, two ball valves are placed into the circuit (see Figure 9). The first ball valve serves primarily for simulating a pipe rupture. If this ball valve is opened, part of the water starts passing through the second branch and the third water meter. The second ball valve regulates the pressure in the testing circuit to achieve the recommended operating conditions of the individual water meters.

**Figure 10:**
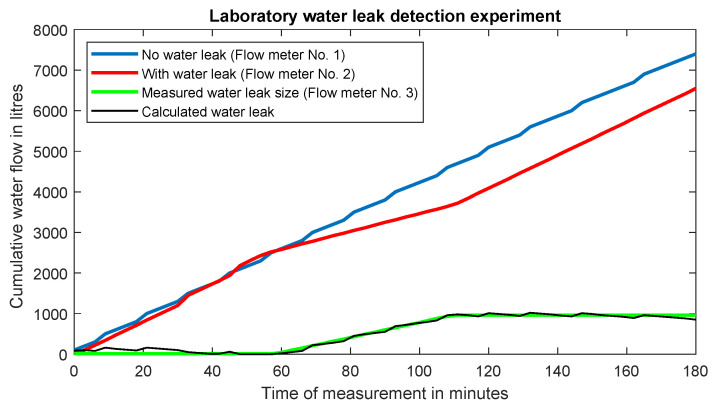
Water leak detection experiment.

Figure 10 shows a time course of a laboratory experiment detecting a possible water leak during a failure. The blue line indicates measurement with no water leak detected, which means that the flow through water meter no. 1 is the same as through water meter no. 2. The red line represents a simulation of failure and pipe rupture in the time of 60:00. The overall simulated water leak is sensed using water meter no. 3, indicated by green colour. The water leak simulation was stopped in the time of 1:49, and the total water leak reached 960 L. The threshold ability to detect water leaks was stated as 100 L, which can be lowered to 10 L by using another type of pulse output. In real operation, in the Czech Republic, this method is used for transporting water over long distances, wherein there can be up to 4 pumping stations on the route (a water meter is installed for each station). Subsequently, during the distribution of drinking water, a so-called foot water meter is installed in each municipality, which measures the water consumption of the entire municipality. Of course, the possibilities of water leak detection in this manner also have their limits (for example, in the case of circular water mains, where the water can be pumped back).

The green line in the graph represents the total measured size of the water leak (water meter no. 3) and the black curve represents the calculated size of the water leak (based on the difference between the values measured on water meter no. 1 and water meter no. 2). Until no leakage is detected, its value is still 0. If a leak is detected, the estimated water leak size is displayed. If the leak is stopped, the total amount of water loss is still displayed, but the curve does not continue to grow (until another water leak is detected). Ideally, when there is no water leakage, the red (water meter no. 2) and blue (water meter no. 1) curves overlap. If a leak is detected, the red curve begins to decrease and the difference between the red and blue curves is equal to the magnitude of the water leak detected.

### 3.1. Signal Coverage Test

**Figure 11:**
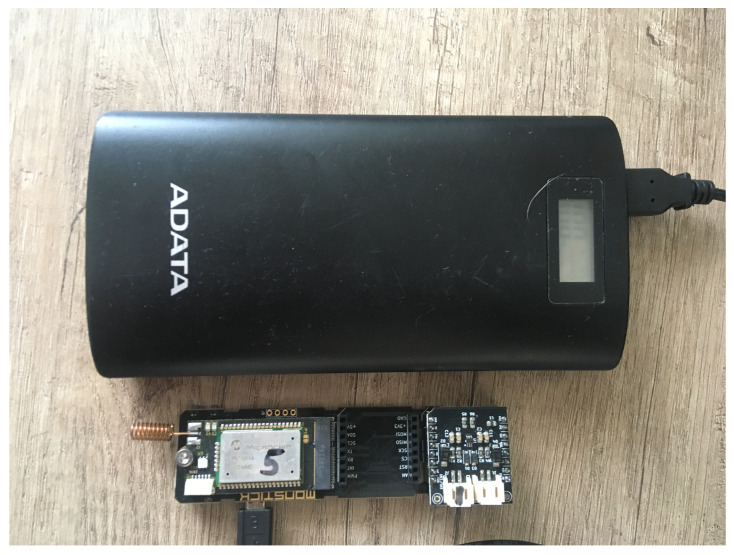
Mobile measuring system composed of modular sensor and power bank.

The measurement took place in the village of Zdarna (area of 1.038 ha, 768 inhabitants, 305 buildings in the village, GPS (Global Positioning System) coordinates of 49.4875122 N, 16.6599706 E). The aim was to compare the signal propagation and its radius in the village of Zdarna. A basic version of the proposed Printed Circuit Board (PCB) with an integrated temperature and humidity sensor (Si7006—A20—*I*M) was used, and the ADATA–P20000D 2000 mAh power bank ensured the power supply. The entire device is shown in Figure 11.

The measurement was conducted using the TTN Mapper application. The first step is a registration of the gateway on www.thethingsnetwork.org, where the GPS coordinates, frequency plan, and antenna type are assigned. The second step requires registration into the network defined by the measuring device (in our case MONSTICK no. 5) with the assignment of the EUI identifier. Then, the measurement can be started using the TTN Mapper mobile application. The measurement record collects all data (temperature and humidity) that are captured by the registered gateway, see Table 4.

#### Measurements in Zdarna Village

**Figure 12:**
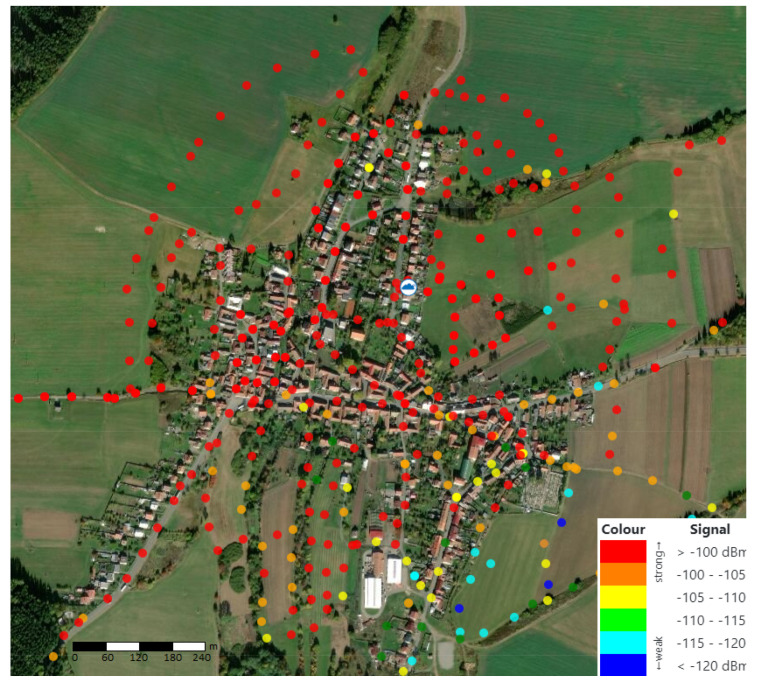
Measurement of the signal strength in Zdarna village (scale 1:6000).

Over 350 measurements were made in the village of Zdarna (see Figure 12), both in a built-up area and at places with potentially new building plots. We found out that dense vegetation can lead to strong signal attenuation, but no locations without signal coverage were identified.

**Figure 13:**
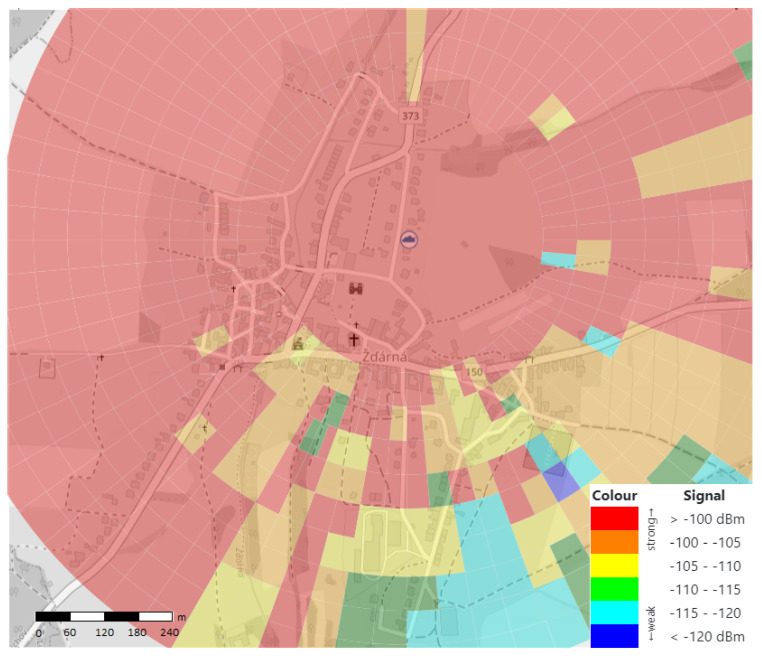
Heat map of the signal coverage in scale 1:6000.

A weak signal was measured only on the village outskirts, in the southern and south-eastern directions. Here, the signal strength ranged from 115 to 120 dBm due to the elevation and relatively high density of buildings. However, these signal values do not prevent a successful data transmission. The associated HEAT MAP, describing the signal transmission in this area, is shown in Figure 13. Coverage in the non-completely measured area is calculated using interpolation.

The cloud icon in the middle of the image is used to indicate the position of the LoRaWAN gateway (GPS coordinates of 49.4704200 N, 16.7590344 E). The maximum distance of the measured point (the building in the village, which is the furthest from our gate) for data transmission is 870 m, when the signal strength at this location reached −113 dBm with the SRN value of 10 dB.

### 3.2. Measurement in Real Conditions

**Figure 14:**
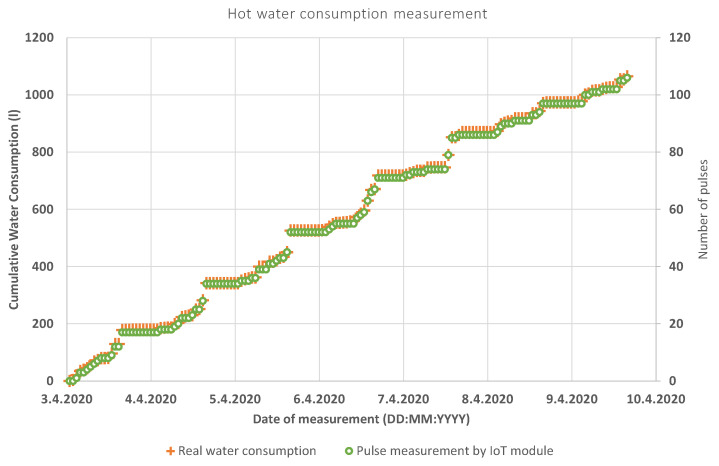
Comparison between remote and manual reading—hot water.

**Figure 15:**
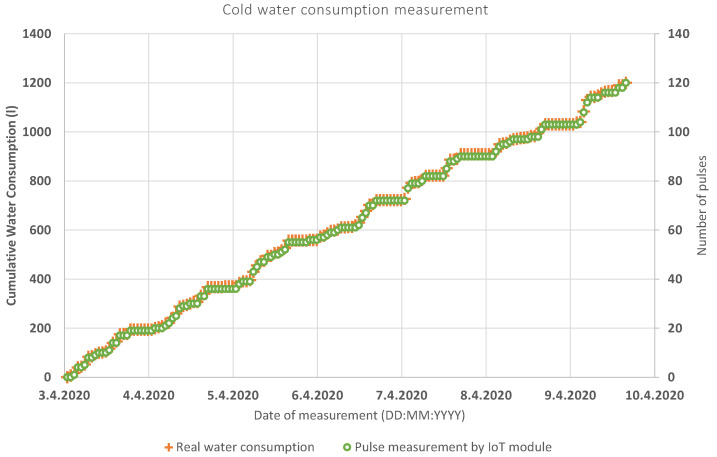
Comparison between remote and manual reading—cold water.

The functionality of the system was verified by reading the cold and hot water in a low-rise apartment building. The results were written down manually to validate the accuracy of the measurement and are shown in Figure 14 (hot water) and Figure 15 (cold water).

The frequency of the automatic and manual data collection was set to 1 h. Subsequently, the data was manually paired, according to the timestamp. This timestamp contained every outgoing message in the following format: YYYY—MM—DD HH:MM:SS. The method of detecting water leaks that has been used is the so-called MNF method (minimum night flow), widely used in water supply. This is the flow that runs into a certain zone/measuring district during the night, when most legitimate water consumption is at its minimum. The period of the minimum night flow usually occurs between two and four o’clock in the morning. In our case (measurement of water consumption in an apartment in a low-rise building), the MNF period was set from 23:59 to 6:00, when the threshold value was set to 10 L. If the flow in the system exceeds this value, a water leak is assumed.

**Figure 16:**
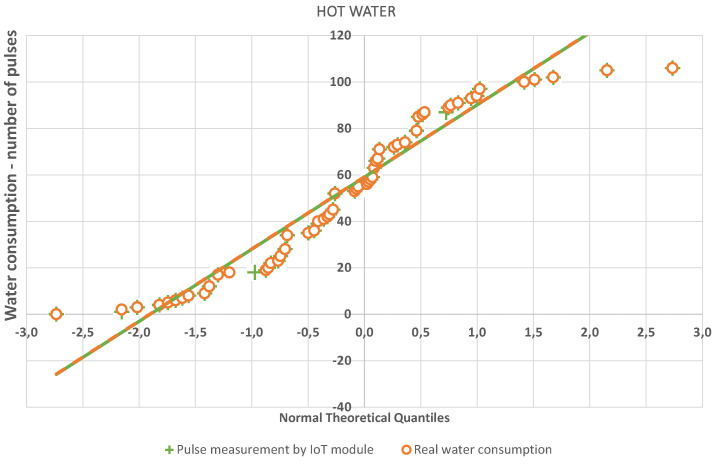
Q-Q plot—hot water.

**Figure 17:**
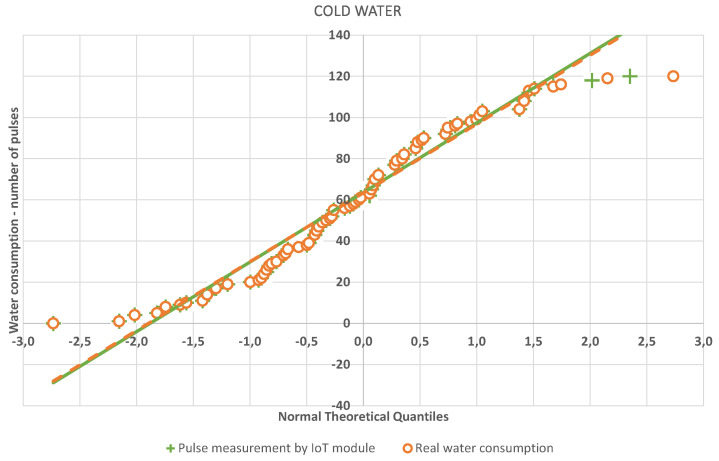
Q-Q plot—cold water.

The quantile-quantile or q-q plot is an exploratory graphical device used to check the validity of a distributional assumption for a data set. If the data really follow the assumed distribution, then the points on the q-q plot will fall approximately on a straight line. The q-q plot provides a visual comparison of the sample quantiles to the corresponding theoretical quantiles. In general, if the points in a q-q plot depart from a straight line, then the assumed distribution is called into question. The q-q plots may be thought of as being “probability graph paper” that makes a plot of the ordered data values into a straight line [72]. As can be seen in Figure 16 and Figure 17, the resulting linear regression lines overlap each other. Thus, both measurement methods have the same probability densities. The method of measurement by means of an IoT sensor is thus relevant for practical application.

## 4. Discussion

Since the weather conditions constantly worsen and the Smart City project develops, we can expect an increasing emphasis on sustainable water management, smart measuring of consumption, and the detection of water leaks. The development of these technologies could positively affect the behaviour of the individual consumers because the final user can have a detailed outline in their household about their consumption. In case of the failure (pipe rupture, leak, running toilets or water taps), a warning message is generated, which could reduce not only the possible leaks, but also the resulting financial burden. This fact is supported by the EU thanks to Directive 2018/2002 of the European Parliament and the Council, introducing mandatory monthly billing for thermal energy costs.

Three low-power technologies for data transmission from water meters to the users are nowadays suitable for use in longer distances; LoraWAN, Sigfox, and NarrowBand IoT. We can expect increasingly massive deployment of these smart technologies due to the major development of IoT technologies and the succession of 5G networks. Our platform is ready to integrate these technologies thanks to the modular solution, which does not require the use of a particular water meter type and allows water companies to choose different techniques and producers.

Two approaches to remote water meter readings are available; machine vision and pulse output. The former is not suitable for wet-running (flood) water meters because of probable fogging or watering the glass, which prevents image recognition. On the contrary, the latter can be enabled under any conditions, and is less energy demanding, so it can be used without electricity.

Our solution enables water companies to connect multiple types of sensors for monitoring the ambient temperature, flood sensors, or to secure the access to the shaft using a magnetic detector, providing an informative message about the access to the area. Our system also includes the LoraWAN gateway, which allows us to cover the wide area. Therefore, considerable funds could be saved. Local operators charge fees for connecting each device to the network when the price is in the range from 0.5 to 1.5 USD, so using a higher number of sensors could be very expensive. Moreover, coverage by the desired technology may not be available in the selected location. Deployment of the low-cost gateway can be considered as an ideal solution. However, it still requires basic infrastructure in the form of access to the Ethernet network and ensuring POE power.

The main advantages of our solution include modularity, where multiple sensors can be connected to one device (for example, a flood sensor, a hatch opening or/and another water meter). This is useful, for example, in the case of housing units that have water meters for both hot and cold water. Thanks to the integrated temperature sensor, we can also detect too low a temperature, which could lead to freezing of the pipeline and the subsequent failure. Thanks to this modularity, our device is also ready for the use in other areas, such as waste management (smart bin) or readings of other types of energy (such as electricity). Thanks to the MNF method, we are also able to detect possible water leaks. However, nowadays there are also solutions that use artificial intelligence based on neural networks to detect water leaks [73,74]. The device is initially in the learning mode, trying to learn the patterns of the users’ behaviour. Nevertheless, at this time, the users are responsible for the reliability and proper operation of the system. More advanced devices are able not to only report an error condition, but also to stop the water supply to the building to prevent further possible leaks. The development of this artificial intelligence seems to be another suitable step to improve our system. It is also important to look at the economic aspects. The price of our equipment is around 40 USD per piece (in the case of buying 100 or more pieces). It can also be assumed that companies will want to pass on these costs to the consumers through the increased water and sewerage charges. However, it should also be noted that water companies will have lower labour costs, since they will not need the staff who now have to conduct the readings manually. There are also other problems associated with this fact. As described above, there is increasing pressure to provide consumers with the most up-to-date information possible and to bill energy consumption more frequently, which would mean hiring new staff to make these readings. Another burning issue is privacy, where the end customers have water meters installed directly in their houses or apartments and the company’s employees must be allowed to enter these buildings. For these reasons, the interest of water companies in these smart solutions is still growing in the Czech Republic.

## 5. Conclusions

The paper deals with the development and deployment of the IoT platform for smart measuring of water consumption and the detection of failures using water leaks. The entire system was designed as modular, wherein the solutions can be found according to the current needs of the desired area and conditions. The LoraWAN technology is used for data transmission, including our LoraWAN gateway. The first part of the paper describes the design and HW properties of the proposed system in detail and focuses on the review of the already existing solutions. The second part describes the actual measurement and the testing of the proposed solution under both laboratory and real conditions. The study also includes a heat map of signal propagation in the rural and urban areas. Then, the measurement in the apartment unit was conducted using hand-operated reading as well as automatic reading by means of the proposed IoT module. The results showed that the average relative error, when measuring using the IoT module, was under 3%. The weaknesses of the solution were identified and discussed. The discussion also outlined new opportunities that future research can take due to the continuous innovation in IoT technologies.

## Figures and Tables

**Table 1:** Basic properties of the STM32L431RB processor.

Chip type	STM32L431RB
SRAM memory size	64 kB
FLASH memory size	128 kB
Operating voltage	1.71–3.6 V
Core frequency	80 MHz
Pin count	64 pins
Temperature range	−40 ${}^{{}^{\circ}}$C–105 ${}^{{}^{\circ}}$C
I2C	3
UART	3
SPI	3

**Table 2:** Technical specifications of outdoor 868 MHz antenna.

Frequency Range (MHz)	824 MHz–960 MHz
Bandwidth (MHz)	70
Gain (dBi)	10
VSWR kHz	<=1.5
Input Impedance ( Ω)	50
Polarization	Vertical
Maximum input power (W)	50
Lightning protection	DC Ground
Input connector type	N Female
Operating temperature (${}^{{}^{\circ}}$C)	−40–60

**Table 3:** LoRaWAN EU863-870 TX Data rate [69].

Data Rate	Configuration	Approximate Physical Bit Rate (bit/s)
0	SF12/125 kHz	250
1	SF11/125 kHz	440
2	SF10/125 kHz	980
3	SF9/125 kHz	1760
4	SF8/125 kHz	3125
5	SF7/125 kHz	5470

**Table 4:** Example of measurement record using TTN mapper.

Date and Time	2020–04–21 @ 09:28
Node	MONSTICK5
Gateway ID	40D63CFFFE1F4309
Location accuracy	10.00
Packet ID	14508882
Distance	212.0 m
Data rate	SF12BW125
Frequency	867.7 MHz
RSSI (Received Signal Strength Indication)	−76.0
SNR ( Signal-to-noise ratio)	9.8
Altitude	641.4 m

## Abbreviations

AMR	Automatic meter reading
BW	Bandwidth
CNN	Convolutional Neural Network
CR	Coding Rate
FSK	Frequency-Shift Keying
GPS	Global Positioning System
IoT	Internet of Things
I2C	Inter-Integrated Circuit
LPWAN	Low-Power Wide-Area Network
MCU	Microcontroller unit
MNF	Minimal Night Flow
NB-IoT	NarrowBand-Internet of Things
PCB	Printed circuit board
PLC	Programmable logic controller
RSSI	Received Signal Strength Indication
SF	Spreading Factor
SMD	Surface Mount Device
SPI	Serial Peripheral Interface
SNR	Signal-to-noise ratio
SWD	Serial Wire Debug
UART	Universal asynchronous receiver-transmitter
